# Biocatalytic Properties and Structural Analysis of Eugenol Oxidase from *Rhodococcus jostii* RHA1: A Versatile Oxidative Biocatalyst

**DOI:** 10.1002/cbic.201600148

**Published:** 2016-06-07

**Authors:** Quoc‐Thai Nguyen, Gonzalo de Gonzalo, Claudia Binda, Ana Rioz‐Martínez, Andrea Mattevi, Marco W. Fraaije

**Affiliations:** ^1^ Molecular EnzymologyGroningen Biomolecular Sciences andBiotechnology InstituteUniversity of GroningenNijenborgh 49747 AGGroningenNL; ^2^ Departmento de Química OrgánicaUniversidad de Sevillac/Profesor García González 141012SevillaSpain; ^3^ Department of Biology and BiotechnologyUniversity of PaviaVia Ferrata 127100PaviaItaly; ^4^ Stratingh Institute for ChemistryUniversity of GroningenNijenborgh 49747 AGGroningenNL

**Keywords:** biocatalysis, dehydrogenation, enzyme structures, kinetic resolution, oxidases

## Abstract

Eugenol oxidase (EUGO) from *Rhodococcus jostii* RHA1 had previously been shown to convert only a limited set of phenolic compounds. In this study, we have explored the biocatalytic potential of this flavoprotein oxidase, resulting in a broadened substrate scope and a deeper insight into its structural properties. In addition to the oxidation of vanillyl alcohol and the hydroxylation of eugenol, EUGO can efficiently catalyze the dehydrogenation of various phenolic ketones and the selective oxidation of a racemic secondary alcohol—4‐(1‐hydroxyethyl)‐2‐methoxyphenol. EUGO was also found to perform the kinetic resolution of a racemic secondary alcohol. Crystal structures of the enzyme in complexes with isoeugenol, coniferyl alcohol, vanillin, and benzoate have been determined. The catalytic center is a remarkable solvent‐inaccessible cavity on the *si* side of the flavin cofactor. Structural comparison with vanillyl alcohol oxidase from *Penicillium simplicissimum* highlights a few localized changes that correlate with the selectivity of EUGO for phenolic substrates bearing relatively small *p*‐substituents while tolerating *o*‐methoxy substituents.

## Introduction

Biocatalysts have become a valuable tool for performing selective oxidation reactions that usually require harsh conditions and reagents when performed by conventional chemistry approaches.^1^, ^2^ Oxidases make up an emerging biocatalyst class for carrying out biotransformations that involve two‐ or four‐electron oxidations of substrates with use of molecular oxygen as a mild oxidant.^3^ Among them, the so‐called flavoprotein oxidases can be valuable for synthetic applications, because they can participate in a broad variety of oxidative procedures with high chemo‐, regio‐, and/or enantioselectivity.^4^, ^5^, ^6^ Catalysis for these enzymes occurs in two half‐reactions: 1) flavin cofactor reduction by oxidation of the substrate, and 2) reoxidation of the flavin by molecular oxygen.^7^ A wide set of flavoprotein oxidases have been extensively employed in biotransformations, with glucose oxidase,^8^ d‐amino acid oxidase,^9^ and cholesterol oxidase^10^ as well‐known examples.

The vanillyl alcohol oxidase (VAO) family is a group of flavoprotein oxidases that share a common FAD‐binding domain and present similar structural features.^11^, ^12^, ^13^ In addition to alcohol oxidations, leading in most cases to aldehydes and ketones, the enzymes of this family can also catalyze amine oxidations, thiol oxidations, hydroxylations, and even C−C bond‐formation reactions.^5^ A representative family member is VAO from *Penicillium simplicissimum*, an enzyme that acts on 4‐hydroxybenzylic compounds.^14^, ^15^ Kinetic studies have shown that the action of VAO involves an initial hydride transfer from the substrate to the FAD; this is facilitated by residues of the protein and generates a *p*‐quinone methide intermediate. Afterwards, the reduced flavin is reoxidized by molecular oxygen, yielding hydrogen peroxide, whereas the *p*‐quinone methide undergoes hydration in the VAO active site, yielding the corresponding alcohol or aldehyde.^16^, ^17^ However, for some substrates the hydration of the *p*‐quinone methide is inefficient, resulting in the formation of the corresponding *p*‐alkenylphenol: that is, formally, a dehydrogenation process.^18^

The genome of *Rhodococcus jostii* RHA1 contains a remarkably large number of genes coding for oxidative enzymes.^19^ Among them, one gene codes for a eugenol oxidase (EUGO) that displays significant sequence identity with VAO (≈40 %) and a similar but not fully overlapping pattern of substrate preferences.^20^ Unlike VAO, which expresses poorly in *Escherichia coli*, recombinant EUGO can be produced in large quantities (a rather impressive 160 mg of pure protein from 1 L of *E. coli* culture), which makes it an attractive target for large‐scale oxidative transformations.

In this report, the EUGO‐catalyzed oxidation of eugenol and vanillyl alcohol, the dehydrogenation of various α,β‐unsaturated ketones, and the kinetic resolution of a racemic secondary alcohol were examined. Optimal conditions for these biocatalytic conversions were determined, revealing that the enzyme is quite tolerant towards organic solvents. Valuable insights into the substrate selectivity of EUGO were obtained by resolving enzyme crystal structures in complexes with isoeugenol, coniferyl alcohol, vanillin, and benzoate at 1.7–2.6 Å resolution. Inspection of the active‐site cavity structure reveals a conserved substrate‐binding mode with respect to that of VAO but also highlights specific features that fine‐tune substrate specificity.

## Results and Discussion

### Substrate specificity of eugenol oxidase

**Scheme 1:**
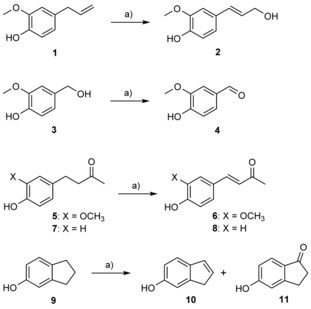
Reactions catalyzed by EUGO from *R. jostii* RHA1. a) EUGO, Tris**⋅**HCl (50 mm, pH 7.5), DMSO, RT, 200 rpm.

Previous experiments had shown that EUGO is active on phenolic substrates, with the identification of eugenol, vanillyl alcohol, vanillylamine, and indan‐5‐ol as good substrates (*k*_cat_=0.26–12 s^−1^), whereas 4‐ethylguaiacol and 4‐(methoxymethyl)phenol were poorly converted (*k*_cat_=0.004–0.026 s^−1^).^20^ Although it was shown that EUGO displays oxidase activity towards these aromatic compounds, no product analysis had been carried out for any of the above substrates. Therefore, as a first step in our project, we performed conversions and subsequent product analyses to establish which phenolic products can be prepared with the aid of EUGO (Scheme 1). Incubation of eugenol (**1**) with the enzyme in Tris**⋅**HCl buffer (pH 8.0) containing 1 % (*v/v*) DMSO afforded coniferyl alcohol (**2**) with complete conversion after 4 h at room temperature. If the reaction was performed in the presence of 10 % DMSO (*v/v*) as cosolvent, an increase in the enzymatic activity was observed, with only 2 h being required to reach complete conversion. Vanillyl alcohol (**3**) was very effectively converted by EUGO. After 4 h, vanillin (**4**) was recovered with complete conversion when the oxidation was performed at pH 8.0 either in aqueous medium or in buffer containing 10 % (*v/v*) DMSO. To explore the potential of EUGO we also tested other substrates.

An interesting result was observed when zingerone (**5**), a ginger‐derived natural product,^21^ was incubated with EUGO in Tris**⋅**HCl buffer (pH 8.0). After 4 h, 58 % conversion was observed, with dehydrozingerone (**6**) being obtained as the single product. When the reaction was carried out for 12 h, complete conversion was achieved. Again, the presence of 10 % (*v/v*) DMSO in the reaction medium increased the enzymatic activity, with the reaction being complete after 8 h. This shows that EUGO is also able to catalyze dehydrogenation processes. Interestingly, dehydrozingerone is also present in ginger and exhibits interesting medicinal properties.^22^

Analogously, raspberry ketone (**7**) was incubated with EUGO at room temperature. This led to the formation of 4‐phenylbut‐3‐en‐2‐one (**8**) as the sole dehydrogenation product. The enzyme presented significantly lower activity with this compound than with zingerone, with only 48 % conversion being observed after 24 h at pH 8.0.

Previous experiments have shown that indan‐5‐ol (**9**) is a EUGO substrate,^20^ which is interesting in view of the cyclic substituent of this compound. After 24 h incubation of this substrate at room temperature and pH 8.0, 34 % conversion was achieved. The resulting mixture contained two different products in a 7:3 proportion. Analysis by MS/GC and NMR revealed that the major product was 1*H*‐inden‐6‐ol (**10**), the dehydrogenation product, whereas 5‐hydroxyindan‐1‐one (**11**) was obtained as the minor product. Again, when the oxidation was performed in the presence of 10 % (*v/v*) DMSO, the enzymatic activity was doubled, leading to 63 % conversion after 24 h.

Other potential substrates, such as 4‐(1,3‐dithiolan‐2‐yl)phenol, capsaicin, (4‐hydroxyphenyl)acetonitrile, 3‐(4‐hydroxyphenyl)propanoic acid, 2‐methoxy‐4‐methylphenol, and 4‐(hept‐1‐enyl)‐2‐methoxyphenol, did not show any significant oxidase activity nor yield any product after incubation with EUGO for 24 h.

These data were complemented by measurements of the initial oxidase rates for some of the EUGO substrates (see Table S1 in the Supporting Information). Next to vanillyl alcohol, eugenol (**1**) and its analogue 4‐(1‐hydroxyethyl)‐2‐methoxyphenol (**12**) were found to be the best substrates for this biocatalyst (2.1 and 4.3 s^−1^, respectively), whereas raspberry ketone (**7**) was by far the most slowly reacting substrate (0.02 s^−1^) of all tested. In essence, EUGO prefers phenolic substrates with *ortho*‐methoxy substituents and a relatively small group at the *para* position.

### Enzymatic dehydrogenation of raspberry ketone (7): Effect of organic cosolvent, temperature, and pH on EUGO activity

The data above show that raspberry ketone (**7**) was only slowly converted by EUGO. For this reason, we set out to optimize the reaction conditions using **7** as a substrate. Because we had observed that EUGO is quite tolerant towards DMSO [10 % (*v/v*) of DMSO resulted in higher levels of conversion for all tested substrates], several solvents were tested in the enzymatic dehydrogenation of this ketone. The reaction carried out in the presence of 10 % (*v/v*) DMSO afforded the final product **8** with a slightly higher level of conversion than achieved in the aqueous medium (60 % conversion after 24 h; Table 1, entry 2). The use of other cosolvents, with different physicochemical properties, led to much lower levels of conversion (entries 3–7).

**Table 1:** Effect of organic cosolvents, temperature, and pH on EUGO activity in the catalysis of the dehydrogenation of raspberry ketone (**7**) under different reaction conditions after 24 h.

	Cosolvent (*v/v*)	pH	*T* [°C]	*c* [%]^[a]^
1	none	8.0	25	48
2	10 % DMSO	8.0	25	60
3	10 % 1,4‐dioxane	8.0	25	10
4	10 % CH_3_CN	8.0	25	≤3
5	10 % EtOAc	8.0	25	17
6	10 % CH_2_Cl_2_	8.0	25	≤3
7	10 % *t*BuOMe	8.0	25	7
8	10 % DMSO	8.0	17	15
9	10 % DMSO	8.0	30	43
10	10 % DMSO	8.0	37	38
11	10 % DMSO	8.0	45	33
12	10 % DMSO	8.0	60	≤3
13	10 % DMSO	7.0	25	23
14	10 % DMSO	7.5	25	38
15	10 % DMSO	9.0	25	53
16	10 % DMSO	10.0	25	57

[a] Conversion determined by GC.

**Figure 1:**
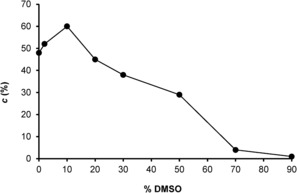
Effect of DMSO concentration on the degree of conversion (•) in EUGO‐catalyzed oxidation of raspberry ketone (**7**, 2.0 mm) after 24 h.

With DMSO selected as the best cosolvent for the enzyme, the effect of its concentration on the enzymatic activity was analyzed (Figure 1). The conversion of **7** catalyzed by EUGO is optimal at 10 % (*v/v*) cosolvent. Higher DMSO concentrations led to progressive deactivation of the biocatalyst, although it is noteworthy that a moderate level of conversion (30 %) was still obtained at 50 % (*v/v*) DMSO after 24 h. This shows that EUGO is fairly solvent‐tolerant.

The effect of temperature on the EUGO‐catalyzed bioconversion of **7** in Tris**⋅**HCl (pH 8.0)/10 % (*v/v*) DMSO medium was also analyzed (Table 1, entries 8–12). At 17 °C, only 15 % conversion was obtained after 24 h. Higher temperatures led to increased levels of conversion, with 60 % of **8** being achieved at 25 °C. A slightly lower level of conversion was observed on working at higher temperatures but EUGO remained active at 45 °C, with 33 % conversion after 24 h. In contrast, no reaction was observed at 60 °C.

Dehydrogenation of raspberry ketone was also performed in Tris**⋅**HCl buffer with 10 % (*v/v*) DMSO at different pH values (from pH 7.0 to 10.0; entries 13–16). The enzyme showed higher activity at higher pH values (from 8.0 to 10.0), with levels of conversion around 50–60 % after 24 h. Lower pH values led to a rapid decrease in conversion, with only 23 % conversion at pH 7.0.

Collectively, this set of experiments indicated that EUGO is highly solvent‐tolerant and maximally active at room temperature, whereas it is rather sensitive to variation of the pH of the medium.

### Eugenol oxidase stability

EUGO is a robust enzyme, being active over a wide range of pH values and tolerating the use of several cosolvents. We determined its thermostability by determining the apparent melting temperatures (*T*_m_ values) under different conditions by the ThermoFAD method.^23^ At pH values from 5.0 to 8.0 the enzyme is exceptionally thermostable, exhibiting *T*_m_ values around 65 °C (Table S2). Moreover, the presence of 10 % (*v/v*) DMSO or ethyl acetate does not significantly affect the enzyme's thermostability. Only extreme pH conditions or the addition of some organic cosolvents decrease the *T*_m_ below 60 °C. This high thermostability is in line with the observation that EUGO can be employed in reaction mixtures including an organic cosolvent. As would be expected, enzyme‐stabilizing agents such as glycerol and salts such as NaCl have a positive effect on the thermostability of EUGO.

### Kinetic resolution of 4‐(1‐hydroxyethyl)‐2‐methoxyphenol [(±)‐12] catalyzed by EUGO

**Scheme 2:**
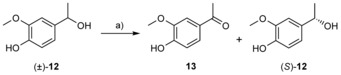
EUGO‐catalyzed kinetic resolution of racemic 4‐(1‐hydroxyethyl)‐2‐methoxyphenol [(±)‐**12**]. a) EUGO, Tris**⋅**HCl (50 mm, pH 7.5), cosolvent, 30 °C, 200 rpm.

Because vanillyl alcohol [**3**] is an excellent substrate for EUGO, its racemic analogue 4‐(1‐hydroxyethyl)‐2‐methoxyphenol [(±)‐**12**] was studied in a secondary alcohol kinetic resolution procedure (Scheme 2). In this process, one enantiomer of the substrate is oxidized to the ketone while the other enantiomer hence becomes enantiomerically enriched. EUGO is a true oxidase, so molecular oxygen consumption leads to the formation of hydrogen peroxide. For this reason, the reactions were performed in the presence of catalase, which catalyzes the decomposition of hydrogen peroxide, thereby regenerating molecular oxygen. It was observed that the presence of small amounts of catalase indeed led to slightly higher levels of conversion with no effect on the selectivity (data not shown). Racemic (±)‐**12** was found to be selectively oxidized by EUGO into ketone **13**, as shown in Table 2. Initial experiments performed with a Tris**⋅**HCl buffer (50 mm, pH 9.0) showed that the kinetic resolution occurred rapidly and with very low selectivity (Table 2, entry 1). Use of lower pH values (see pH 7.5, entry 2) led to significantly higher enantioselectivity (*E=*35), resulting in the enantioenrichment of (*S*)‐**12**. Conversely, the presence of 10 % (*v/v*) DMSO in the reaction medium decreased the level of conversion and the selectivity of the reaction (entry 3). For this reason, the amount of this organic cosolvent was reduced to 5 % (*v/v*), resulting in a higher level of conversion, as shown in entry 4.

**Table 2:** EUGO‐catalyzed kinetic resolution of the racemic alcohol 4‐(1‐hydroxyethyl)‐2‐methoxyphenol [(±)‐**12**].^[a]^

	Cosolvent	*t* [h]	*c* [%]^[b]^	*ee* [%]^[c]^	*E* ^[d]^
1^[e]^	none	4	49	23	2.0
2	none	4	33	45	35
3	10 % DMSO	4	29	17	3.0
4	5 % DMSO	2	30	35	14
5	5 % EtOAc	4	31	49	38
6	5 % *t*BuOMe	1.5	40	55	18
7	5 % *i*Pr_2_O	4	39	59	45
8	5 % hexane	4	48	13	1.5
9	5 % CH_2_Cl_2_	4	≤3	–	–
10	5 % toluene	4	6	12	3.7
11	5 % octan‐2‐ol	1.5	50	65	9.1

[a] For reaction conditions, see the Experimental Section. [b] Determined by GC. [c] Determined by HPLC. [d] Enantiomeric ratio, *E*=ln[(1−*c*)/(1−*ee*_s_)]/ln[(1−*c*)/(1+*ee*_s_)].^24^ [e] Reaction performed at pH 9.0.

The effects of alternative organic cosolvents (5 %) in the EUGO‐catalyzed kinetic resolution of (±)‐**12** were studied (entries 5–11). The use of ethyl acetate (Table 2, entry 5) led to similar results in terms of activity and selectivity, whereas the best result was afforded with *i*Pr_2_O, in a process with a promising selectivity (*E*=45) and a 39 % level of conversion after 4 h (entry 7). Kinetic resolutions in reaction media containing 5 % (*v/v*) *t*BuOMe, hexane, or octan‐2‐ol were much faster, with up to 50 % conversion after 1.5 h, but the selectivity was from moderate to poor. Oxidations with cosolvents such as toluene or CH_2_Cl_2_ occurred with very low conversion efficiencies (entries 9 and 10).

### The overall three‐dimensional structure of EUGO

**Figure 2:**
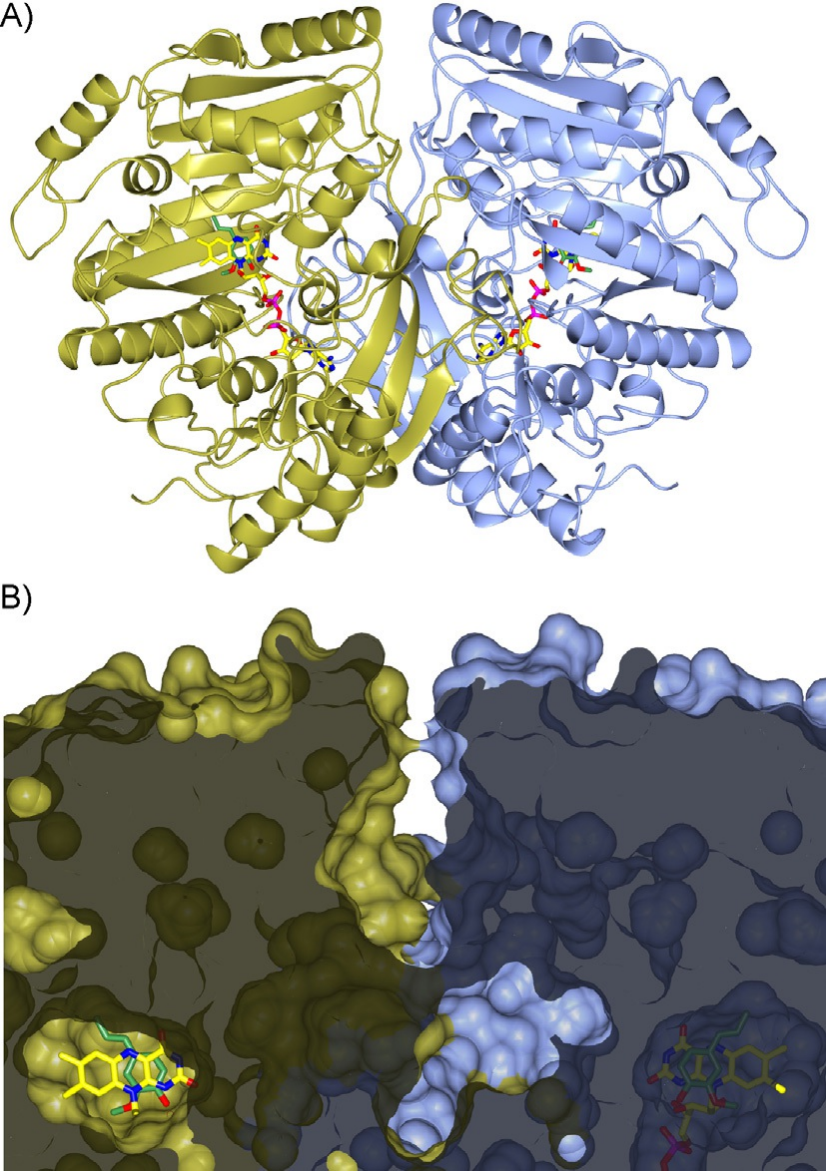
Crystal structure of eugenol oxidase from *R. jostii* RHA1 in complex with the inhibitor isoeugenol. A) Ribbon diagram of the EUGO dimer with the two monomers in ice blue (monomer A) and gold (monomer B), respectively. B) Close‐up sliced view of the EUGO surface in the vicinity of the catalytic pocket. The inhibitor isoeugenol is buried in a pocket in close contact with a deep invagination originating at the top of the dimer and being formed by residues at the dimer interface, which might represent the pathway for ligand access to the active site. The orientation and atom coloring is the same as that of the structure shown in (A). The FAD carbon atoms are shown in yellow, the ligand carbon atoms in lawn green, oxygen atoms in red, nitrogen atoms in blue, and phosphorus atoms in magenta.

**Figure 3:**
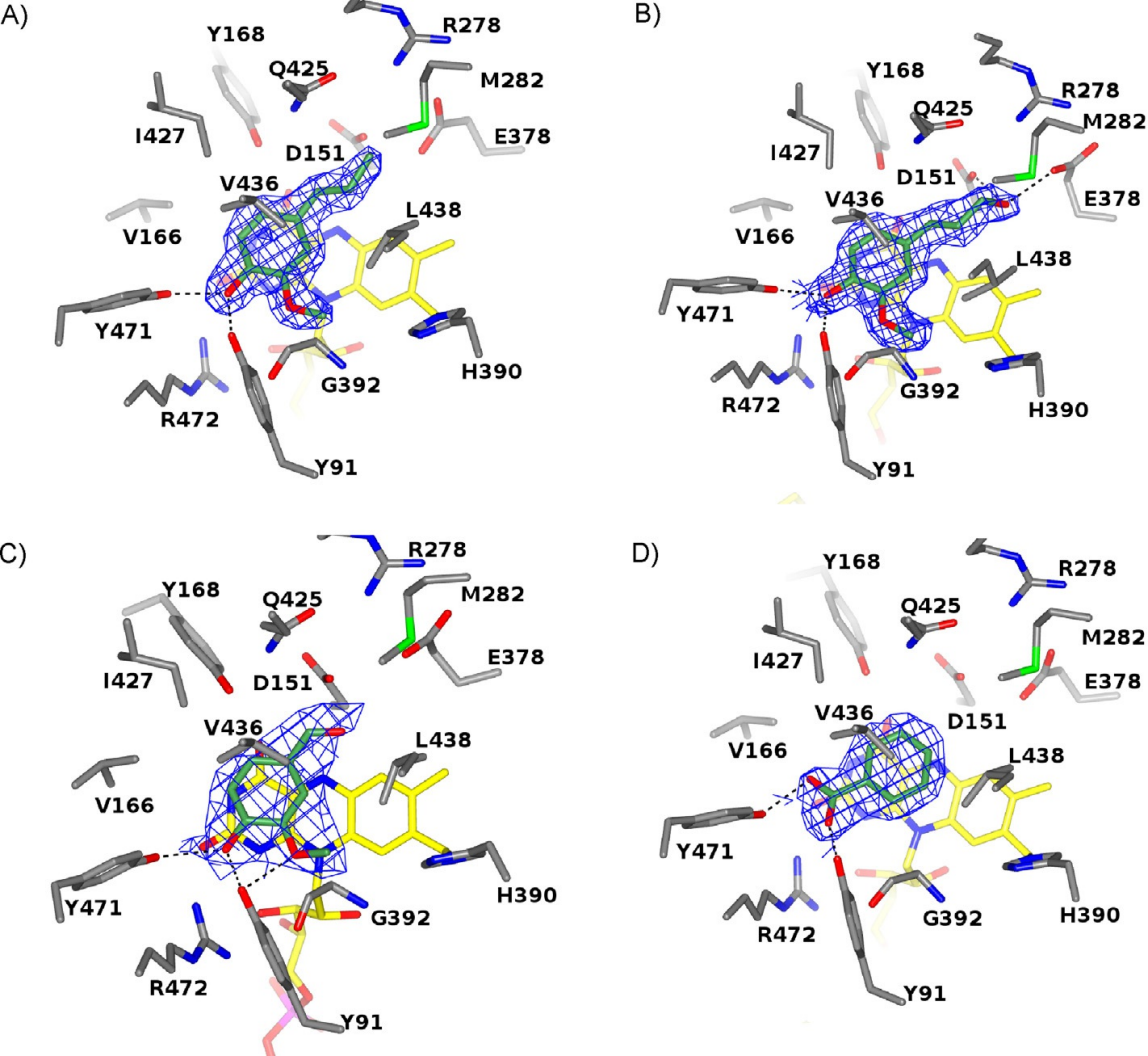
The EUGO active site in complex with A) isoeugenol, B) coniferyl alcohol, C) vanillin, and D) benzoate. Unbiased 2*F*_o_−*F*_c_ omit electron density maps calculated at 1.7–2.6 Å resolution and contoured at 1.0 *σ* are drawn in chicken‐wire style. Hydrogen bond contacts are shown as dashed lines. Residues in direct contact with the bound ligands are labeled. Protein, ligand, and FAD carbon atoms are colored in gray, lawn green, and yellow, respectively, oxygen atoms are in red, nitrogen atoms are in blue, and phosphorus atoms are in magenta. The orientation is the same as that of monomer A in Figure 2.

The structure of EUGO in complex with the inhibitor isoeugenol was solved at 1.7 Å resolution by molecular replacement with use of the VAO monomer (PDB ID: 2VAO)^11^ as a search model. The asymmetric unit contains two enzyme subunits forming a compact dimer that is also observed in solution (Figure 2 A and B). The dimer interface area is quite large, approximately 3500 Å^2^, accounting for 16.5 % of the monomer's surface as calculated by PISA.^25^ The overall structure of EUGO is highly similar to that of VAO, as indicated by a 1.1 Å rmsd for 490 Cα atom pairs (45 % sequence identity).^11^ As predicted, EUGO also shares with VAO the presence of a characteristic covalent bond between His390 (equivalent to His422 of VAO) and the C8M atom of the flavin ring (Figure 3). Nevertheless, the oligomeric organization in the two enzymes is partly different, because, though both form essentially the same functional dimer (1.3 Å rmsd for 999 Cα atom pairs), EUGO lacks the dimer–dimer interacting loop that in VAO mediates the octameric structure of the enzyme.

### The active site of EUGO

The active site of EUGO consists of a round‐shaped cavity located in front of the flavin cofactor (Figure 2 B). As in VAO, this substrate‐binding site is completely solvent‐inaccessible. However, inspection of the dimeric structure of EUGO reveals a putative admission path for the ligands. The active‐site cavity of each monomer is in close contact with a funnel‐shaped large chamber that runs along the dimer interface to the top of the structure and might represent a passageway for the diffusion of the substrate into the binding site (in the orientation of Figure 2 B). The structural bases of substrate and inhibitor binding by the cavity were explored by solving the crystal structures of EUGO in complexes with isoeugenol (inhibitor), coniferyl alcohol (**2**, reaction product and inhibitor), and vanillin (**4**, reaction product); these were obtained by crystal soaking, resulting in highly defined electron density maps (Figure 3 A–C).

All these ligands bind with their aromatic moiety stacking against the pyrimidine ring of the flavin and the Cα atom of the aliphatic substituent lying right above the flavin N5 atom at a distance of 3.2 Å. In this orientation, the ligand 4‐hydroxy group is H‐bonded to Tyr91 and Tyr471 and is also in close contact with Arg472 (3.5 Å). These three residues are involved in the stabilization of the phenolate form of the substrate and are strictly conserved in VAO. The fact that such a Tyr‐Tyr‐Arg cluster forms an anion‐binding site is supported by the structure obtained from native crystals (i.e., not soaked in a ligand solution), which exhibited a clear electron density peak close to the flavin rings of both monomers present in the asymmetric unit. This residual density was tentatively assigned to a benzoate ion bound with its carboxylate group directly interacting with the Tyr‐Tyr‐Arg residues (Figure 3 D). Indeed, we found benzoate to be a weak EUGO inhibitor, probably captured by the enzyme in the cell and retained during purification. Collectively, these features are fully consistent with the substrate being bound in the phenolic form and oxidized through a reaction mechanism involving a hydride transfer from the Cα to the flavin N5, coupled to the stabilization of a *p*‐quinone methide intermediate as demonstrated for VAO.^16^

**Figure 4:**
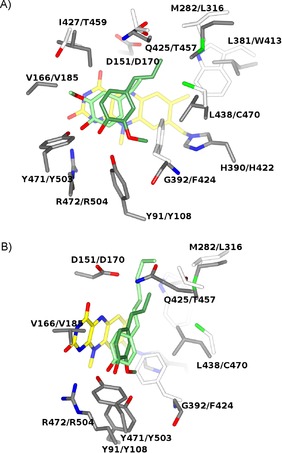
Comparison between EUGO and VAO active sites. A) The substrate‐binding site of EUGO in complex with isoeugenol (lawn green) is superposed onto that of VAO in complex with the same ligand (light green; PDB ID: 2VAO). Unconserved residues in VAO are drawn with carbon atoms in white. The orientation and atom coloring is the same as that of the structure shown in Figure 2. For the sake of clarity, Tyr168, Arg278, and Glu378 were omitted in this figure. The γ carbon atom of isoeugenol in the VAO structure was disordered and was not included in the refined model.^11^ B) The superposition of the EUGO**⋅**isoeugenol structure onto VAO in complex with 4‐(hept‐1‐enyl)phenol (light green; PDB ID: 1AHZ) shows that EUGO features a slightly narrower cavity due to the presence of Gln425 (Thr457 in VAO), which would clash with ligands with longer alkyl chains. In this figure the structure is rotated by about 45° with respect to (A). For the sake of clarity, the side chains of I427/T459 as well as the labels of L381/W413 and H390/H422 were omitted.

However, comparative analysis of the three‐dimensional structures also revealed striking differences between EUGO and VAO. Above all, inhibitor/product binding differs in a 180° flipped orientation. This can be visualized by superimposing the isoeugenol complexes of the two enzymes. As shown in Figure 4 A, the ligand methoxy groups are in opposed orientations and occupy different niches in the active sites of the two enzymes. In EUGO, the methoxy moiety is in close contact with Gly392, which replaces the Phe424 residue of VAO. This bulkier aromatic side chain of VAO likely creates steric hindrance, impeding the binding orientation found in EUGO. Conversely, the flipped isoeugenol bound to VAO positions the methoxy group in direct contact with a valine residue that is conserved in EUGO (Val166, Figure 4 A).

It is gratifying that these findings correlate with the non‐overlapping patterns of substrate preferences and selectivities displayed by the two enzymes. Indeed, inspired by these structural data, we tested 4‐allyl‐2,6‐dimethoxyphenol. This dimethoxy compound is not a substrate of VAO.^15^ Conversely, we found that it is converted by EUGO with moderate efficiency (*k*_cat_=0.49 s^−1^, *K*_m_=0.8 μm). Thus, the presence of Gly392 (instead of Phe424 in VAO) is a key feature that enables EUGO to act on molecules bearing two *o*‐methoxy substituents. This is in contrast with VAO, which does not accept dimethoxy‐substituted molecules as substrates.^15^

The other relevant element in EUGO that partially differs from the situation in VAO involves the section of the active‐site cavity that is involved in the binding of the substrate alkyl chain. Here, despite a set of conserved residues (Asp151, Tyr168, Arg278, and Glu378, corresponding to Asp170, Tyr187, Arg312, and Glu410 in VAO), there are a few major differing amino acids between the two enzymes: Thr457, Trp413, and Leu316 in VAO are replaced by Gln425, Leu381, and Met282, respectively, in EUGO (Figure 4 B). In particular, these replacements collectively reshape this part of the substrate site, which becomes narrower in EUGO than in VAO. As a result, it is more difficult for EUGO to accept long aliphatic chains and hydrophobic substituents than it is for VAO (Figure 4 B). Indeed, whereas VAO is reactive towards alkylphenols and can accommodate aliphatic substituents containing up to seven carbon atoms,^11^, ^26^ we found no activity of EUGO with such compounds.

## Conclusion

The synthetic potential of eugenol oxidase (EUGO) in oxidative procedures has been demonstrated. The data show that EUGO is a robust biocatalyst, tolerating a wide range of organic solvents and high temperatures. Interestingly, the addition of DMSO as cosolvent increased the enzymatic activity in most of the catalytic processes. EUGO could also be employed in the kinetic resolution of a racemic secondary alcohol with moderate to good selectivity. Structural analysis of the EUGO catalytic center showed that the enzyme shares a generally conserved substrate binding mode with VAO but with key different residues finely controlling the substrate specificity. With an effective expression system, detailed structural information, and generic oxidase screening methods to hand, EUGO appears a perfect candidate for engineering tailored oxidase variants.

## Experimental Section

**General materials and methods**: Recombinant eugenol oxidase from *R. jostii* RHA1 was overexpressed and purified according to previously described methods.^20^ All other chemicals and solvents were obtained from Acros Organics, Alfa Aesar, ABCR, Sigma–Aldrich, TCI Europe, and Roche Diagnostics. Flash chromatography was performed with Merck silica gel 60 (230–400 mesh). ^1^H NMR, ^13^C NMR, and DEPT spectra were recorded with TMS (tetramethylsilane) as the internal standard, with a Bruker AC‐300‐DPX (^1^H: 300.13 MHz and ^13^C: 75.4 MHz) spectrometer. Mass spectra were recorded by ESI^+^ with a HP1100 chromatograph mass detector or by EI with a Finnigan MAT 95 spectrometer. GC/MS analysis for compounds **1**–**11** was performed with a GC Hewlett Packard 6890 Series II and a Hewlett Packard 5973 chromatograph MS (Agilent Technologies) equipped with a HP‐1 cross‐linked methyl siloxane column (30 m×0.32 mm×0.25 μm, 1.0 bar N_2_). The injector temperature was 225 °C, and the flame ionization detector (FID) temperature was 250 °C. The following temperature program was employed: 100 °C (5 min), 10 °C min^−1^ to 250 °C (2 min). *t*_R_ **1**: 11.0 min. *t*_R_ **2**: 15.6 min. *t*_R_ **3**: 12.2 min. *t*_R_ **4**: 11.3 min, *t*_R_ **5**: 14.5 min. *t*_R_ **6**: 16.4 min. *t*_R_ **7**: 11.8 min. *t*_R_ **8**: 12.9 min, *t*_R_ **9**: 10.6 min. *t*_R_ **10**: 10.8 min. *t*_R_ **11**: 9.6 min. GC analysis for measuring the conversion of (±)‐**12** was performed with a Restek RtβDEXse (30 m×0.25 mm×0.25 μm, 1.0 bar N_2_) column. The following temperature program was used: 70 °C (5 min), at 3 °C min^−1^ to 160 °C (2 min), 20 °C min^−1^ to 180 °C (7 min). *t*_R_ **12**: 41.4 min. *t*_R_ **13**: 40.8 min. To monitor levels of conversion, substrates and products were quantified by use of calibration curves. HPLC analyses were developed with a Hewlett Packard 1100 LC liquid chromatograph. The following conditions were used for the determination of the enantiomeric excess of alcohol **12**: Chiralcel OJ‐H column (0.46×25 cm), isocratic eluent: *n*‐hexane/EtOH (95:5), 40 °C, flow 1 mL min^−1^. *t*_R_ (*S*)‐**12**: 31.4 min. *t*_R_ (*R*)‐**12**: 35.1 min.

**General procedure for the enzymatic oxidations catalyzed by isolated EUGO**: Unless stated otherwise, starting compounds **1**, **3**, **5**, **7**, and **9** (2–10 mm) were dissolved in Tris**⋅**HCl buffer (50 mm, pH 8.0, 1.0 mL) containing, when indicated, organic cosolvent (10 %, *v/v*) and EUGO (0.33 μm). Reaction mixtures were shaken at 250 rpm and room temperature for the times indicated. Once reaction was complete, the crude mixtures were extracted with EtOAc (3×500 μL). The organic phases were dried onto Na_2_SO_4_ and analyzed directly by GC/MS.

**Kinetic resolution of racemic 4‐(1‐hydroxyethyl)‐2‐methoxyphenol catalyzed by EUGO**: Unless stated otherwise, the starting racemic alcohol (±)‐**12** (10 mm, Scheme 2) was dissolved in Tris**⋅**HCl buffer (50 mm, pH 7.5) containing, when stated, organic cosolvent (5–10 %, *v/v*, 1.0 mL), catalase (2 μL, six units μL^−1^), and EUGO (1.0 μm). Reaction mixtures were shaken at 250 rpm and 30 °C in a rotatory shaker for the times indicated. Once reaction was complete, the crude mixtures were extracted with EtOAc (2×500 μL). The organic phases were dried onto Na_2_SO_4_ and analyzed directly by GC and HPLC in order to determine the levels of conversion to **13** and the enantiomeric excesses of the alcohol (*S*)‐**12**.

**Measurement of initial rates**: Conversion rates were determined by monitoring the consumption of molecular oxygen. Oxygen concentrations were monitored by use of a REDFLASH sensor spot in a 3 mL cuvette in combination with a Firesting O_2_ detector and light source (Pyroscience, Aachen, Germany). Measurements were performed with substrates (2.0 mm) in KPi buffer (50 mm, pH 7.5) containing DMSO (10 %, *v/v*).

**Determination of kinetic parameters for 4‐allyl‐2,6‐dimethoxyphenol**: The reaction was monitored by following the absorbance of the product at 270 nm (*ϵ*=14.1 mm ^−1^ cm^−1^ at pH 7.5) in Tris**⋅**HCl (50 mm, pH 7.5) at 25 °C.

**Analysis of EUGO thermostability**: EUGO thermostability was assayed by use of the ThermoFAD protocol,^23^ with a MiniOpticon real‐time PCR detection system and 48‐well RT‐PCR plates (Biorad Laboratories). Samples were prepared by mixing EUGO (50 μm, 2.0 μL) in Tris**⋅**HCl (10 mm, pH 7.5) together with the buffers and/or organic solvents to a final volume of 20 μL and in duplicate for each condition tested.

**Protein crystallization, X‐ray data collection, and structure determination**: Native EUGO was crystallized by use of the sitting‐drop vapor diffusion technique at 20 °C by mixing equal volumes of EUGO (15 mg mL^−1^) in Tris**⋅**HCl (10 mm, pH 7.5) and mother liquor containing PEG6000 (24 %, *w/v*) and Tris**⋅**HCl (0.1 m, pH 8.0). All EUGO**⋅**ligand complexes were prepared by soaking (0.5–2 h) the crystals in cryoprotectant solutions consisting of PEG6000 (26 %), Tris**⋅**HCl (0.1 m, pH 8.0), glycerol (20 %), and the compound of interest (5.0 mm), followed by flash‐freezing in liquid nitrogen. X‐ray diffraction data were collected at the PXI and PXIII beamlines of the Swiss Light Synchrotron in Villigen, Switzerland (SLS) and at the ID23–1 beamline of the European Synchrotron Radiation Facility in Grenoble, France (ESRF). The images were integrated by MOSFLM,^27^ whereas data scaling was performed by use of programs of the CCP4 suite.^28^ The detailed data processing statistics of the collected datasets are shown in Table S3. EUGO's structure was solved by molecular replacement with use of MOLREP^29^ and the coordinates of VAO (PDB ID: 2VAO)^11^ as the search model devoid of all ligand and water molecules. COOT^30^ and REFMAC5^31^ programs were employed to carry out alternating cycles of model building and refinement (data shown in the Supporting Information). Figures were created by use of CCP4mg.^32^

## Supporting information

As a service to our authors and readers, this journal provides supporting information supplied by the authors. Such materials are peer reviewed and may be re‐organized for online delivery, but are not copy‐edited or typeset. Technical support issues arising from supporting information (other than missing files) should be addressed to the authors.

Supplementary
