# Supplementary material for: Biocatalytic Properties and Structural Analysis of Eugenol Oxidase from Rhodococcus jostii RHA1: A Versatile Oxidative Biocatalyst
Source: Chembiochem. 2016 Jun 7;17(14):1359–66. doi: 10.1002/cbic.201600148 (PMC5089669; doi:10.1002/cbic.201600148)
Supplement: Supplementary file 1 — Supplementary [file CBIC-17-1359-s001.pdf]

CHEMBIOCHEM

Supporting Information

Biocatalytic Properties and Structural Analysis of Eugenol Oxidase from *Rhodococcus jostii* RHA1: A Versatile Oxidative Biocatalyst

Quoc-Thai Nguyen+,[a, c] Gonzalo de Gonzalo+,[b] Claudia Binda,[c] Ana Rioz-Martínez,[d] Andrea Mattevi,*[c] and Marco W. Fraaije*[a]

cbic_201600148_sm_miscellaneous_information.pdf

Table S1. Initial rate values for some of the EUGO substrates when using a 2.0 mM concentration of substrate in a 50 mM KPi buffer pH 7.5 containing 10% $v\ v^{-1}$ DMSO.[a]

Substrate	Rate (s^{-1})
eugenol (**1**)	2.1
vanillyl-alcohol (**3**)	5.1
zingerone (**5)**	0.63
raspberry ketone (**7**)	0.02
5-indanol (**9**)	0.18
4-(hydroxy-1-ethyl)-2-methoxyphenol (**12**)	4.3

Table S2. Melting temperature of EUGO in different conditions determined by *Thermo*FAD method

Entry	Conditions	T_m (°C)	Entry	Conditions	T_m (°C)
1	10 mM Tris/HCl pH 7.5	60.5	11	B-R buffer pH 10.0	51.0
2	50 mM Tris/HCl pH 7.5	61.0	12	10% DMSO, 50 mM Tris/HCl pH 7.5	60.5
3	50 mM HEPES pH 7.5	66.0	13	20% DMSO, 50 mM Tris/HCl pH 7.5	58.5
4	50 mM KPi pH 7.5	65.0	14	10% EtOH, 50 mM Tris/HCl pH 7.5	54.5
5[a]	B-R buffer pH 4.0	57.0	15	10% EtOAc, 50 mM Tris/HCl pH 7.5	61.5
6	B-R buffer pH 5.0	64.5	16	10% ^{i}PrOH, 50 mM Tris/HCl pH 7.5	55.0
7	B-R buffer pH 6.0	67.5	17	10% MeOH, 50 mM Tris/HCl pH 7.5	57.0
8	B-R buffer pH 7.0	65.0	18	10% glycerol, 50 mM Tris/HCl pH 7.5	62.0
9	B-R buffer pH 8.0	69.0	19	100 mM NaCl, 50 mM Tris/HCl pH 7.5	67.0
10	B-R buffer pH 9.0	53.0	20	500 mM NaCl, 50 mM Tris/HCl pH 7.5	67.0

[a] B-R buffer: 40 mM Britton–Robinson buffer prepared by mixing H_3BO_3, H_3PO_4, and CH_3COOH, titrated to the desired pH with NaOH.[2]

Table S3. Data collection and refinement statistics.

	Native	Isoeugenol	Coniferyl alcohol	Vanillin
		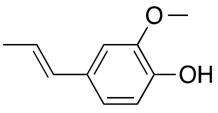	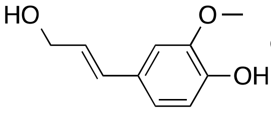	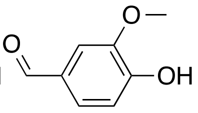
PDB Code	5FXF	5FXD	5FXE	5FXP
Space group	$P2_12_12_1$	$P2_12_12_1$	$P2_12_12_1$	$P2_12_12_1$
Resolution (Å)	1.9	1.7	1.9	2.6
a, b, c (Å)	57.3, 96.9, 179.7	57.7, 96.7, 179.7	57.5, 97.2, 179.1	57.92, 97.5, 180.2
R_{sym} [a,b] (%)	14.9 (98.3)	12.0 (99.0)	13.3 (69.0)	14.1 (41.6)
Completeness[b] (%)	99.5 (99.6)	98.7 (98.0)	97.2 (97.6)	91.6 (80.4)
Unique reflections	79217	109694	77427	29395
Redundancy[b]	4.7 (4.9)	3.9 (3.5)	3.8 (3.3)	3.9 (2.7)
I/σ[b]	8.6 (2.7)	7.5 (1.8)	6.2 (1.7)	7.7 (2.2)
Number of atoms:				
protein	8232	8249	8247	8246
FAD/ligand/water	2×53/2×9/304	2×53/2×12/627	2×53/2×13/478	2×53/2×10/30
Average B value for ligand atoms ($Å^2$)	23.1	21.2	17.7	28.8
R_{cryst}[b,c] (%)	16.5 (22.6)	16.1 (25.9)	16.2 (28.4)	17.9 (26.2)
R_{free}[b,c] (%)	21.2 (26.7)	19.9 (28.7)	21.1 (28.5)	26.0 (35.1)
Rms bond length (Å)	0.019	0.019	0.019	0.011
Rms bond angles (°)	1.98	1.94	1.98	1.98

[a] $R_{sym}=\sum|I_i - \langle I\rangle|/\sum I_i$, where I_i is the intensity of ith observation and $\langle I\rangle$ is the mean intensity of the reflection.

[b] Values in parentheses are for reflections in the highest resolution shell.

[c] $R_{cryst}=\sum|F_{obs} - F_{calc}|/\sum|F_{obs}|$ where F_{obs} and F_{calc} are the observed and calculated structure factor amplitudes, respectively. R_{cryst} and R_{free} were calculated using the working and test sets, respectively

References

[1] J. Jin, H. Mazon, R. H. van den Heuvel, D. B. Janssen, M. W. Fraaije, *FEBS J*. **2007**, *274*, 2311–2321.

[2] H. T. S. Britton, R. A. Robinson, *J. Chem. Soc.* **1931**, *0*, 1456–1462.
